# Ultrafast diffusion of Ionic Liquids Confined in Carbon Nanotubes

**DOI:** 10.1038/srep28518

**Published:** 2016-06-23

**Authors:** Aziz Ghoufi, Anthony Szymczyk, Patrice Malfreyt

**Affiliations:** 1 Institut de Physique de Rennes, IPR, CNRS-Université de Rennes 1, UMR CNRS 6251, 35042 Rennes, France; 2 Institut des Sciences Chimiques de Rennes, UMR 6226 CNRS, Université de Rennes 1, 263 Avenue du Général Leclerc, 35042 Rennes, France; 3 Institut de Chimie de Clermont-Ferrand, ICCF, UMR CNRS 6296, Université Clermont Auvergne, Université Blaise Pascal, BP 10448, 63000 Clermont-Ferrand, France

## Abstract

Over the past decade many works have focused on various aspects of the dynamics of liquids confined at the nanoscale such as e.g. water flow enhancement through carbon nanotubes (CNTs). Transport of room temperature ionic liquids (RTILs) through various nanochannels has also been explored and some conflicting findings about their translational dynamics have been reported. In this work, we focus on translational dynamics of RTILs confined in various CNTs. By means of molecular dynamics simulations we highlight a substantially enhanced diffusion of confined RTILs with an increase up to two orders of magnitude with respect to bulk-phase properties. This ultrafast diffusion of RTILs inside CNTs is shown to result from the combination of various factors such as low friction, molecular stacking, size, helicity, curvature and cooperative dynamics effects.

Room Temperature Ionic Liquids (RTILs) are molten salts composed of large organic cations and inorganic or organic anions. This class of solvents has been widely studied over the past decades due to their important applications^1^,^2^ associated with their unique properties (negligible vapor pressure, thermal stability, non-flammability, high ionic conductivity and wide electrochemical stability window). More recently, the confinement of RTILs at the nanoscale has gained much attention given their relevance to important industrial applications such as electrochemical double-layer capacitors (EDLCs)^1^,^2^,^3^,^4^,^5^ and dye-sensitized solar cells (DSSCs) for solar energy conversion^6^,^7^,^8^. RTILs have also been considered as potential candidates as additives in lubricants^9^ and ionogels^10^ for which immobilization of RTILs inside nanoporous systems (carbon nanotubes (CNTs)^11^, silica frameworks^12^…) is required. The macroscopic performance of nanoconfined RTILs is ruled by both their structural and dynamic properties at the nanoscale^13^,^14^,^15^, which are strongly modified with respect to the bulk phase. Understanding the behavior of RTILs under nanoconfinement is then critical for the rational design of EDLCs, DSSCs and ionogels with optimal properties. While structural and electrical properties have been largely investigated translational dynamics of confined RTILs remains controversial and this lack of knowledge strongly limits our understanding of confined RTILs behavior.

Several numerical and theoretical works have focused on RTILs confined into cylindrical nanopores, such as CNTs^14^,^16^,^17^,^18^,^19^,^20^,^21^,^22^ or silica materials^23^,^24^,^25^, and slit-like pores such as rutile slabs^26^ or graphitic pores^13^. Results from these studies suggest that both local RTIL structure and local dynamics of confined cations and anions are very complex and heterogeneous, depending strongly on the distance between ions and the pore wall. In addition to their tremendous physical properties, CNTs represent models of hydrophobic nanopores enabling to explore ultraconfinement effects on local structure and dynamics of liquids. In this context, some interesting properties were reported for various liquids confined into CNTs such as fast diffusion^21^,^27^,^28^, low friction^27^,^28^, superpermittivity^29^, local surdensity^21^,^27^,^29^,^30^, etc. Dynamics of confined RTILs remains unclear and is still a matter of debate. By means of molecular dynamics (MD) simulations Pinilla *et al*. evidenced a faster molecular motion of [dmim^+^][Cl^−^] confined between two parallel structureless walls, notably for ions located close to the walls^8^. Using quasielastic neutron scattering Chathoth *et al*. obtained an enhanced diffusivity for [bmim^+^][Tf_2_N^−^] and [H_2_NC(dma)_2_][BETI] confined into mesoporous carbons^31^,^32^. Their results provided the first experimental observations of RTILs diffusing faster under confinement than in the bulk phase. Hung and coworkers reported MD simulations of varying amounts of [Emim^+^][TFMSI^−^] confined inside uncharged slit-like graphitic nanopores of different widths^13^. The local dynamics of [Emim^+^][TfMSI^−^] was found heterogeneous and strongly dependent on the distance between ions and the pore walls. Dynamics of ions located in the center of a slit pore of 5.02 nm in width was found similar to that of the bulk RTIL. Recently, Li *et al*. carried out MD simulations of [C_4_mim^+^][Tf_2_N^−^] confined in silica and carbon mesopores and observed a slowdown in ion diffusion inside both mesopores compared with bulk values^23^.

In the present study, we explore the interplay between translational dynamics and structure of RTILs confined into uncharged CNTs with the aim of addressing several fundamental questions: i) How is RTIL translational dynamics impacted inside hydrophobic CNTs? ii) Is there translational heterogeneity according to the distance between ions and the CNT wall? iii) Is there any connection between local structure and dynamics? iv) Are there some cooperative effects? v) What is the impact of the CNT topology on RTIL dynamics? To address these issues, MD simulations of three RTILs, [C_4_mim^+^][Tf_2_N^−^], [C_8_isoq^+^][Tf_2_N^−^] and [Me_3_BuN^+^][Tf_2_N^−^] confined in (i) two armchair CNTs of radius 13.05 Å (CNT(20,20)) and 27 Å (CNT(40,40)), (ii) a zigzag CNT of radius 13.05 Å (CNT(34,0)) and (iii) a slit pore made of graphene sheets separated by a distance of 27 Å were carried out. Models, force fields and computational procedure are detailed in the Method Section. [C_8_isoq^+^][Tf_2_N^−^] and [Me_3_BuN^+^][Tf_2_N^−^] were chosen so as to investigate the impact of aromatic rings on RTIL translational dynamics.

## Results

**Figure 1:**
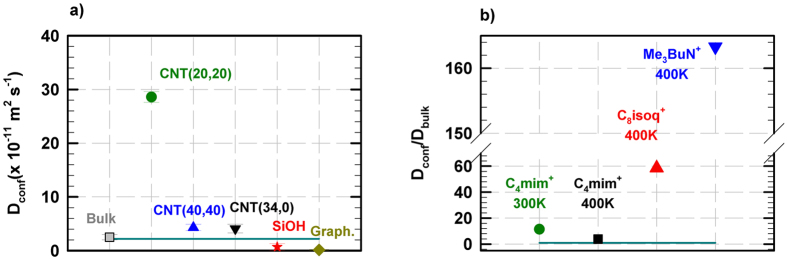
(**a**) Diffusion coefficient of [C_4_mim^+^] confined in different nanopores (D_conf_) at 300 K and 1 bar. The straight line corresponds to the experimental value. (**b**) Ratio between the confined diffusion coefficient (D_conf_) and the bulk diffusion coefficient (D_bulk_) for the three RTILs confined in the CNT(20,20). The straight line corresponds to D_conf_ = D_bulk_.

We report in Fig. 1a the cation diffusion coefficient for RTIL [C_4_mim^+^][Tf_2_N^−^] confined in different nanopores. Details about diffusion coefficient calculation are provided in the Method Section. A substantial increase in the diffusion coefficient of [C_4_mim^+^] confined in the CNT(20,20) was observed with respect to the bulk value. Indeed, as shown in Fig. 1b the diffusion coefficient of [C_4_mim^+^] at 300 K is tenfold higher in the CNT(20,20) than in the bulk phase. Our results are qualitatively in line with those obtained by Pinilla *et al*. who showed a local faster molecular motion of [dmim^+^][Cl^−^] confined between two parallel structureless walls^8^. Our results are also in good agreement with recent experiments performed by Zanotti and coworkers who have shown an enhanced [Omim^+^][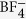] mobility induced in CNTs^33^.It is worth noting that bulk values inferred from simulations were found in very good agreement with experiments^23^,^31^,^34^ and faster translational dynamics of [C_4_mim^+^] with respect to [Tf_2_N^−^] in the bulk phase^34^,^35^ was well reproduced by MD simulations (see Table 1). Moreover, as shown in the Supplementary (Figure S2) two regimes with different dynamics were highlighted, a subdiffusive slow regime between 00 and 0.01 ns and a fast diffusive behavior from 0.01 to 200 ns. This result is in fair agreement with recent quasi elastic neutron scattering experiments^31^. We also computed the diffusion coefficient of confined [C_4_mim^+^][Tf_2_N^−^] in the (CNT(20,20)) at 400 K so as to evaluate the impact of temperature. As indicated in Table 1 the enhanced diffusion under confinement is still observed but to a lesser extent compared with results obtained at 300 K (the increase in [C_4_mim^+^] diffusion coefficient is about 300% at 400 K against 1000% at 300 K). This impact of temperature on the increase in the diffusion coefficient of confined RTILs was also observed experimentally^31^. These nice agreements between experiments and MD simulations give us confidence in the accuracy of the force field used to describe [C_4_mim^+^][Tf_2_N^−^].

As shown in Fig. 1b and Table 1 the increase in the translational dynamics under nanoconfinement was also observed for both cations and anions of the other two RTILs ([C_8_isoq^+^][Tf_2_N^−^] and [Me_3_BuN^+^][Tf_2_N^−^]). Let us note that different force fields were used to describe the different RTILs, namely all-atom and united-atom force fields (see Method Section), which shows that the enhanced diffusion phenomenon was predicted independently of the force field. Moreover, Fig. 1b shows that the increase in the diffusion coefficient of both [C_8_isoq^+^] and [Me_3_BuN^+^] cations confined in the CNT(20,20) is greater than for [C_4_mim^+^] (in the case of [Me_3_BuN^+^] the increase in the diffusion coefficient with respect to the bulk phase can be as high as 160 at 400 K). These findings suggest that the enhanced diffusion phenomenon is not correlated with the number of aromatic rings in the RTIL structure. Moreover, in this work we studied three RTILs with different sizes and ultrafast diffusion was always evidenced. Let us mention that very recent experiments highlighted an increase in diffusion of other RTILs confined in CNTs. Therefore, although diffusion is quantitatively impacted by the size of RTIL ions the enhanced diffusion phenomenon reported in this work is expected to occur irrespective of ion size^33^.

In contrast to what is observed in the bulk phase, RTIL cations and anions exhibit similar diffusion coefficients when confined in the CNT(20,20) (see Table 1). Such an outcome suggests concerted dynamics under nanoconfinement. It has been reported that translational dynamics of confined species depends on the distance from the pore wall^36^. In Table 2 we report diffusion coefficients of [C_4_mim^+^] and [Tf_2_N^−^] close to the pore surface (referred to as interfacial zone in Table 2) and in the central part of the pore (referred to as inner zone). Let us note that boundaries of both interfacial and inner zones were determined from the density profiles of the ion center of mass (see Figures S1d and S3 in the Supplementary). As shown in Table 2 ion diffusion was found to be similar in both interfacial and inner zones, thus suggesting cooperative effects from the pore surface to the pore center.

Over the past decade some works reported the ultralow friction between water^27^,^37^,^38^,^39^,^40^,^41^,^42^ or organic solvents^28^ and CNTs. The increase in the diffusion coefficient of water trapped in CNTs was connected to this ultralow friction^41^,^43^, which was further correlated with nanotube curvature inducing incommensurability effects between the filling molecules and the nanotube^27^. More recently, it was shown that coupling between water molecules and longitudinal phonon modes of the nanotube could enhance diffusion of confined water by more than 300%^41^. In order to unravel the origin of enhanced diffusion of RTIL confined within CNTs we carried out MD simulations of [C_4_mim^+^][Tf_2_N^−^] confined within several media. As shown in Fig. 1a and in Table 1 the diffusion coefficient of confined [C_4_mim^+^] strongly depends on the confining medium. Notably, its value inside a silica nanopore of 13 Å in radius (referred to hereafter as SiOH) or between graphene slabs (separated by 27 Å) was found to be lower than the bulk value. On the other hand, the diffusion coefficient of [C_4_mim^+^] confined in CNT(40,40) and CNT(34,0) was found to be higher than in the bulk phase although this increase was less pronounced than for the CNT(20,20). Comparing these different nanopores with the CNT(20,20), as a reference system, enables to investigate the impact of various effects on RTIL translational diffusion, namely size (CNT(40,40), R = 27 Å), helicity (CNT(34,0), R = 13.05 Å), roughness (SiOH, R = 13 Å) and curvature (graphene, H = 27 Å) effects.

## Discussion

### Friction, roughness and molecular stacking

**Figure 2:**
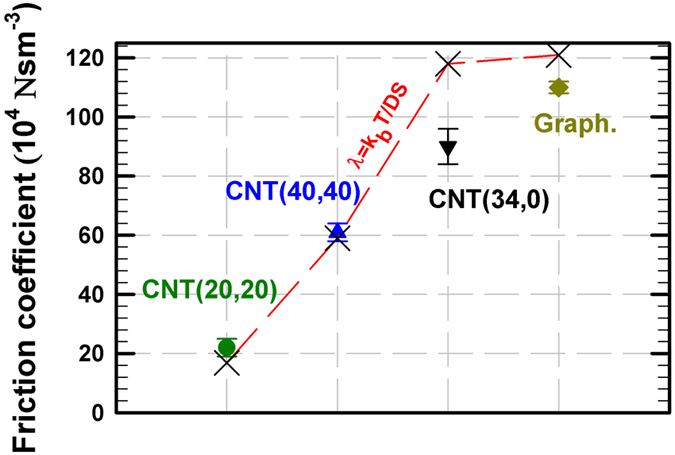
Friction coefficient of [C_4_mim^+^][Tf_2_N^−^] confined in various nanopores at 300 K and 1 bar. The dashed line shows results obtained from the Einstein’s relation.

Based on previous findings on low friction of confined water within CNTs^37^,^38^,^41^,^42^ we first tried to correlate the fast diffusion of RTILs confined in CNTs with low friction. Indeed, a direct correlation between the increase in diffusion coefficient and the decrease in friction is expected according to the Einstein’s relation, i.e. *D* = *k*_*b*_*T*/*Sλ* where *D* is the diffusion coefficient, *k*_*b*_ the Boltzmann’s constant, *T* the temperature, *S* the surface area and *λ* the friction coefficient. Fig. 2 shows the friction coefficient of [C_4_mim^+^][Tf_2_N^−^] confined in the different nanopores (details about the calculation of the friction coefficient are provided in the Method Section). The friction coefficient was found to increase according to the sequence CNT(20,20) < CNT(40,40) < CNT(34,0) < graphene, in agreement with the Einstein’s relation. Furthermore, the results obtained with the CNT(20,20), CNT(40,40) and the graphene slit-like pore highlight a curvature effect already observed for both water and organic molecules confined in CNTs^27^,^28^. Interestingly, the friction was found much higher in the zigzag CNT(34,0) than in the armchair CNT(20,20) although both nanotubes have similar radii, which is in line with the diffusion coefficient obtained in both systems. Thus, it seems that the ultrafast diffusion phenomenon reported in this work is deeply connected to the low friction between RTILs and CNTs.

**Figure 3:**
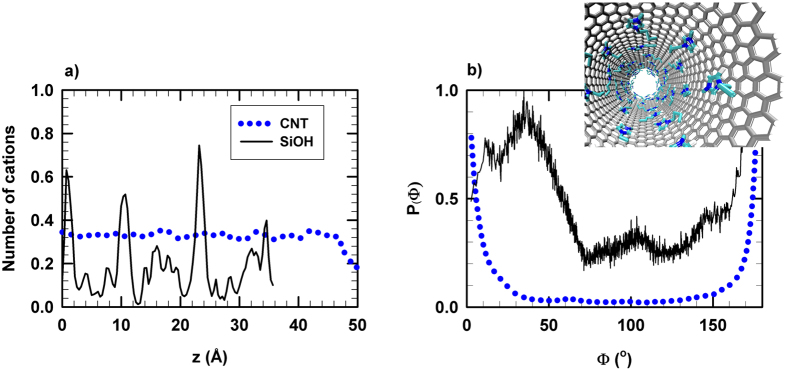
(**a**) Cation axial density (along the *z* axis of nanotube) for [C_4_mim^+^][Tf_2_N^−^] confined in SiOH and CNT(20,20) frameworks at 300 K and 1 bar. (**b**) Distribution of the angle between the axial unit vector of the CNT(20,20) and the [C_4_mim^+^] aromatic ring axis.

Let us note that friction was not computed inside the SiOH nanopore given the absence of decorrelation of forces due to the pore roughness and the strong interfacial anchoring of the RTIL. Indeed, as shown in Fig. 3a the axial density profile of [C_4_mim^+^] inside the SiOH nanopore exhibits several peaks, which highlights RTIL interfacial anchoring while a smooth profile is observed in the CNT(20,20) as a result of surface smoothness and low friction, which promotes free sliding along the nanotube axis. We report in Fig. 3b the distribution of the angle formed by the axis of the [C_4_mim^+^] aromatic ring and that of the nanopore for both SiOH and CNT(20,20) frameworks. As shown in Fig. 3b two maxima were evidenced inside the CNT(20,20), at 0 and 180°, which indicates stacking of RTIL and CNT rings (see snapshot in Fig. 3b) unlike what was observed within the SiOH nanopore. The combination of low friction, surface smoothness and RTIL-CNT stacking could then be at the origin of the enhanced diffusion (RTIL surfing) reported in Fig. 1. Interestingly [Me_3_BuN^+^][Tf_2_N^−^] confined within CNTs also exhibits an ultrafast diffusion (see Fig. 1b) despite the absence of aromatic rings in its structure. As depicted in Figure S4 of the Supplementary the [Me_3_BuN^+^] alkyl chain also adopts a stacking-like configuration with the CNT surface. These findings suggest that *π*-*π* interactions are not responsible for the observed RTIL-CNT stacking phenomenon.

### Size, curvature and helicity effect

**Figure 4:**
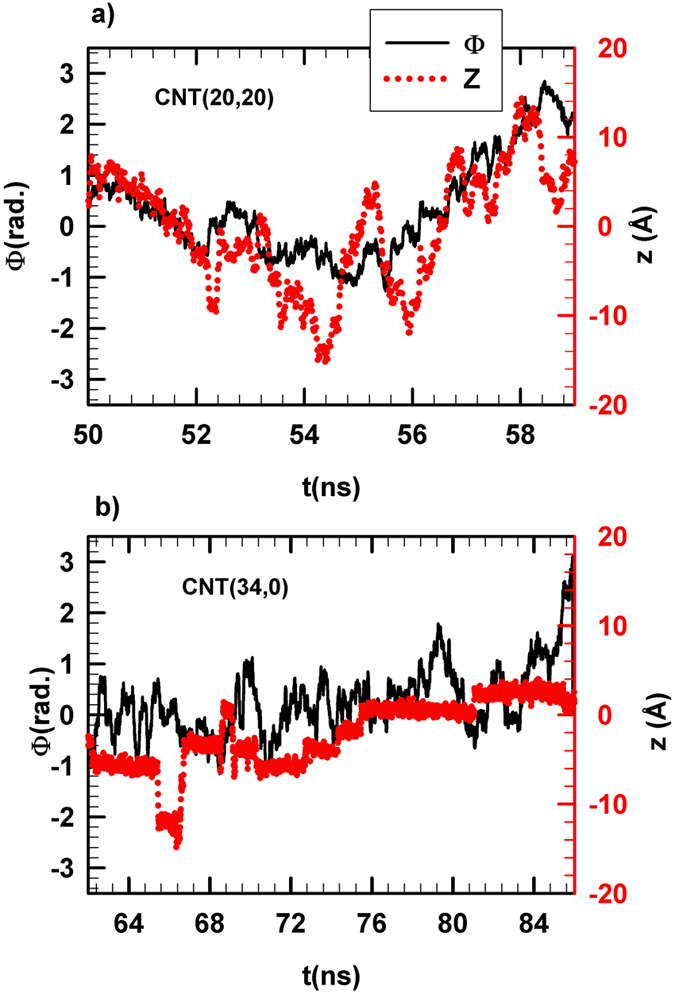
Time evolution of the azimuthal (black and left axis) and axial (red and right axis) coordinates of a single [C_4_mim^+^] cation confined in a) CNT(20,20) and b) CNT(34,0).

As shown in Fig. 1a and Table 1 the RTIL enhanced diffusion phenomenon is weaker inside the CNT(40,40) than inside the CNT(20,20). This pore size effect can be related to the lower friction inside the smaller pore as shown in Fig. 2 (the decrease in friction with CNT radius has also been reported for confined water^27^). In order to disentangle pore size and curvature effects we carried out MD simulations of [C_4_mim^+^][Tf_2_N^−^] confined between two graphene sheets forming a slit pore of 27 Å in width (which corresponds to the CNT(20,20) diameter). As shown in Fig. 1a the enhanced diffusion phenomenon was not observed within the graphene slit-like pore, the [C_4_mim^+^] diffusion coefficient being reduced by about 96% with respect to the bulk phase (Table 1). This outcome is an obvious indication of a strong curvature effect and suggests that different diffusional mechanisms occur within these two pores. The mechanism leading to enhanced diffusion within the CNT(20,20) is highlighted in a movie available in the Supplementary. This latter shows that both translational and azimuthal dynamics are coupled inside the CNT(20,20). We report in Fig. 4a the axial (*z*) and azimuthal (Φ) positions of [C_4_mim^+^] inside the CNT(20,20) as a function of time. An obvious correlation was found between (*z*) and (Φ) thus suggesting a spiral motion inside the nanopore. This correlated motion in both directions is likely to play a significant role in the RTIL enhanced diffusion phenomenon. Such a translational mechanism (spiral diffusion) within CNTs was already observed with small molecules such as water, ethane and ethylene and was attributed to armchair CNT topology inducing preferential helicoidal pathways^44^,^43^. It can be noted that spiral diffusion was also observed within the larger CNT(40,40) (see Figure S5 in the Supplementary) but to a lesser extent than within the CNT(20,20). The impact of CNT helicity was evaluated by comparing RTIL diffusion through the armchair CNT(20,20) and the zigzag CNT(34,0) (both CNTs have identical radius). As shown in Fig. 1a the enhanced diffusion phenomenon was much weaker within the zigzag nanotube than within its armchair counterpart (the [C_4_mim^+^] diffusion coefficient within the CNT(34,0) was found to increase by about 70% with respect to the bulk phase against 1000% for the CNT(20,20)). As shown in Fig. 4b no correlation between [C_4_mim^+^] axial and azimuthal motions was evidenced in the CNT(34,0), thus excluding spiral diffusion. Instead, Fig. 4b suggests that translational diffusion through the zigzag nanotube is discontinuous and characterized by the existence of latency periods during which ions are almost motionless along *z* while keeping azimuthal mobility. These circular motions (instead of helicoidal motions as observed in armchair CNTs) dramatically limit the enhanced diffusion phenomenon inside zigzag CNTs. It is worth noting that surface tension was found similar for both CNTs (6.1 ± 0.9 mN m^−1^ and 8.9 ± 1.4 mN m^−1^ for the CNT(20,20) and the CNT(34,0), respectively) and cannot therefore be invoked to explain the great difference between RTIL translational dynamics within armchair and zigzag nanotubes (details about the calculation of surface tension are provided in the Method Section). Additionally, as shown in Table S1 and Figure S8 (see Supplementary) ion solvation was found almost independent of curvature and CNT helicity.

From a local viewpoint, diffusion coefficients of ions confined in the CNT(20,20) were found identical in both inner and interfacial zones (see Table 2). In other words, the translational motion in the central part of the CNT seems to be induced by ion motion in the interfacial zone. This cooperative effect results from both the cylindrical symmetry and the small size of the CNT(20,20). It is weakened as the pore size increases (see results for the CNT(40,40) in Table 2) and totally vanishes for graphene slabs (slit pore geometry). In this latter case the standard behavior of nanoconfined liquids is recovered, i.e. a decrease in the diffusion coefficient close to the wall (ion diffusion in the interfacial zone of graphene slabs was found to be about 300 times lower than in the inner zone).

To sum up, we reported ultrafast diffusion of RTILs confined in CNT materials. We showed that this enhanced diffusion phenomenon is ruled by an interplay of low-friction, size, helicity, curvature and cooperative dynamics effects. This study clearly highlights the role of pore size that induces cooperative dynamics of confined ionic liquids and the role of pore helicity, curvature and smoothness on the translational dynamics of RTILs. Our results also allow understanding some results reported in the literature such as the local increase in the translational mobility of [Emim^+^][TFMSI^−^] confined inside uncharged slit-like graphitic nanopores^13^ as well as the slowdown of [C_4_mim^+^][Tf_2_N^−^] dynamics when confined in silica and carbon mesopores^23^. These outcomes represent an important piece of work in the fundamental understanding and control of dynamics of ionic liquids confined at the nanoscale. Most RTILs exhibit a nanometric structuration in the bulk phase^45^. Since it generates density fluctuations that reduce ionic mobility, this propensity to self-assembly in transient nanoscopic domains represents an unfavorable condition for ionic conductivity. Thus the use of 2D nanometric confinement within CNTs enables to boost RTIL transport properties. Our results suggest that confinement in a space whose dimension is comparable to the size of the characteristic fluctuation of the systems, could induce considerable changes in RTIL transport properties. Confinement at the nanoscale therefore seems to be a route to increase significantly RTILs ionic conductivity. Further work is needed to explore the impact of the chemical nature of anions (hydrophobicity/hydrophilicity), ion size, temperature and pressure dependence, impurities, etc.

## Methods

### Computational Method and Force Fields

Figure S1 shows the structure of two RTILs under consideration in the present work: butyl-03-methylimidazolium bis (trifluoromethylsulfonyl)imide ([C_4_mim^+^][Tf_2_N^−^]) and octyl isoquinolium bis(trifluoromethyl)sulfonimide ([C_8_isoq^+^][Tf_2_N^−^]). [C_4_mim^+^][Tf_2_N^−^] was modeled from a recent united atom force field^35^. [C_8_isoq^+^][Tf_2_N^−^] was described by means of the non-polarizable AMBER99 force field^46^. Recently, Lisal *et al*. have demonstrated that the generic non-polarizable AMBER force field is able to reproduce RTILs physical properties^47^. This has been corroborated in a recent work where both RTIL interfacial and bulk properties were shown to be well reproduced^48^. A third RTIL, [Me_3_BuN^+^][Tf_2_N^−^], was described by using a revisited OPLS force field^49^. CNT and graphene nanopores were described from a potential developed by Werder *et al*.^50^. A silica nanopore (SiOH) of radius 13 Å was constructed by carving a cylinder in a cubic box of silica. Computational details for surface hydroxylation as well as the force field are provided in ref. 51. Rigid armchair and zigzag-typed CNTs were considered. CNTs with radius R = 13.5 Å (CNT(20,20) and CNT(34,0)) and R = 27 Å (CNT(40,40) were used. The length of all CNTs was set to 100 Å. A graphene-based slit pore made of two graphene sheets (100 × 102 Å) separated by 27 Å was also considered.

All MD simulations were carried out with the DL_POLY package (version 4.0)^52^ using a combination of the velocity-Verlet and the SHAKE-RATTLE algorithms^53^. The Nose-Hoover thermostat^54^,^55^ with a relaxation time of *τ*_*t*_ = 0.5 ps was considered. Periodic boundary conditions were applied in the three directions. MD simulations were performed in the canonical ensemble at T = 300 K and T = 400 K. MD simulations were performed using a time step of 0.002 ps to sample 200 ns (acquisition phase) after a 100 ns equilibration. Electrostatic interactions were truncated at 12 Å and calculated by using the Ewald sum with a precision of 10^−6^. Short range interactions were modeled by using a Lennard-Jones potential and a cutoff of 12 Å. Lennard-Jones interactions between inorganic framework and ionic liquids have been taken into account by means of the Lorentz-Berthelot mixing rule. Statistical errors were estimated using the block average method. Comparisons between bulk and confined properties were performed at 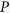 = 1 bar.

Density of confined RTILs was computed by means of an anisotropic barostat^51^ by contacting the empty CNT with two RTIL reservoirs at 1 bar (an example of initial configuration is shown in Figure S1b). Simulation box dimensions were Lx = Ly = 100 Å and Lz = 300 Å. After convergence of the confined RTIL density, MD simulations of a CNT without external reservoirs were carried out (Figure S1c) with simulation box dimensions Lx = Ly = 30 Å and Lz = 100 Å. This configuration was equilibrated in the canonical ensemble for 100 ns.

### Diffusion Coefficient Calculation

Given the radial anisotropy and the cylindrical symmetry the translational diffusion coefficient 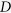 was calculated from the time evolution of the *z* component of the total Mean-Square-Displacement (MSD) of molecular center-of-mass^56^,^57^ according to:


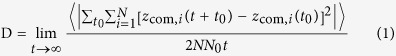


with *t*_0_ the time origin, *N* the number of molecules and *N*_0_ the number of *t*_0_. Note that in the bulk phase the total MSD was considered and was then divided by 6 for determining the bulk diffusion coefficient.

### Friction Calculation

Friction coefficient (*λ*) was computed from Eq. 2 using the correlation of the intermolecular forces between the ionic liquid and the inorganic matrix (*f*)^58^. In Eq. 2 *S* is the surface, *k*_*B*_ the Boltzmann’s constant, *T* the temperature, *N* the number of ions and *N*_*af*_ the number of framework atoms. We report in Figure S7 the correlation function of forces and the friction coefficient for [C_4_mim^+^] [Tf_2_N^−^] in the CNT(20,20). The *λ* values given in the manuscript correspond to the plateau values.


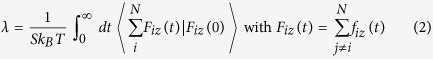


### Surface Tension calculation

Let us note that it was the first time that the surface tension of ionic liquids confined within a nanopore was calculated. Surface tension (*γ*) was calculated by using the non-exponential method^59^ which is based on the usual test-area methodology^60^ where *γ* is expressed as 
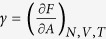
 with *F* the free energy, *A* the surface area, *N* the number of molecules and *V* the volume. Let us note that the non-exponential method was recently developed with a rigorous theoretical background^59^,^61^,^62^,^63^. Thus, the non-exponential approach cannot be considered as an approximation of the usual exponential form (TA). Calculation of 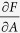 was performed by means of an explicit derivation which provides 
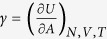
 where *U* is the configurational energy. This expression was approximated through a finite difference such that 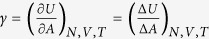 where Δ*U* is the energy difference between two states of different surface areas (Δ*A* = *A*^1^−*A*^0^ where 0 stands for the reference state and 1 stands for the perturbed state). Thus, to maintain the volume constant the following anisotropic transformations were used 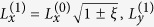 and 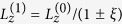 where ξ is the perturbation length as ξ → 0 and *L* the box length. The area of a cylindrical interface is 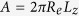 and 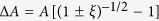, where *R*_*e*_ is the radius of the equimolar dividing surface. *R*_*e*_ was achieved by calculating the radial density profile (*ρ*(*r*)) from 
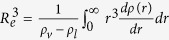
 where *ρ*_*v*_ and *ρ*_*l*_ are the vapor and liquid densities, respectively. Let us note that this choice is arbitrary and other definitions could be used such as a fit of the density profile by means of a hyperbolic function. Indeed, this definition was specifically developed for spherical interfaces. However, we compared the so-calculated *R*_*e*_ with the radius obtained by means of a hyperbolic tangential fit^64^ and only negligible differences were. Therefore, the surface tension can be express as


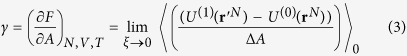


where *U*^(0)^ (**r**^*N*^) and *U*^(1)^(**r′**^*N*^) are the configurational energies of the reference and perturbed states, **r**^*N*^ and **r**′^*N*^ are the configurational space for both states, <…>_0_ stands for that the average taken over the reference state. Surface tensions of [C_4_mim^+^][Tf_2_N^−^] confined in CNT(20,20), CNT(34,0), CNT(40,40) and graphene-based slit pore were 6.1, 8,9 20.9 and 34.4 mN m^−1^ respectively. Error bars were between 1–3 mN m^−1^.

## Additional Information

**How to cite this article**: Ghoufi, A. *et al*. Ultrafast diffusion of Ionic Liquids Confined in Carbon Nanotubes. *Sci. Rep.* **6**, 28518; doi: 10.1038/srep28518 (2016).

## Supplementary Material

Supplementary Video

Supplementary Information

## Figures and Tables

**Table 1:** Diffusion coefficients (in 10^−11^ m^2^ s^−1^) of RTILs at 1 bar and different temperatures.

	Cation	Anion
[C_4_mim^+^][Tf_2_N^−^] (300 K)		
Bulk(sim.)	2.5_2_	1.9_2_
Bulk(exp.)^34^	2.2	1.6
Confined(CNT(20,20))	28.6_9_	28.5_9_
Confined(CNT(40,40))	4.3_2_	4.3_2_
Confined(CNT(34,0))	4.1_2_	4.1_2_
Confined(Graph.)	0.1_01_	0.1_01_
Confined(SiOH)	0.5_08_	0.2_03_
[C_4_mim^+^][Tf_2_N^−^] (400 K)		
Bulk(sim.)	16.5_6_	11.9
Confined(CNT(20,20))	63.6_16_	62.9_16_
[C_8_isoq^+^][Tf_2_N^−^] (400 K)		
Bulk(sim.)	2.6_2_	2.7_2_
Confined(CNT(20,20))	152.4_42_	152.6_42_
[Me_3_BuN^+^][Tf_2_N^−^] (400 K)		
Bulk(sim.)	0.3_01_	0.2_01_
Confined(CNT(20,20))	48.9_12_	48.7_12_

The statistical errors were estimated using the block average method from 5 trajectories. The subscripts give the accuracy of the last decimal(s), e.g. 2.5_8_ means 2.5 ± 0.8 and 63.6_16_ means 63.6 ± 1.6.

**Table 2:** Diffusion coefficients (in 10^−11^ m^2^ s^−1^) of [C_4_mim^+^][Tf_2_N^−^] (300 K, 1 bar) and [C_8_isoq^+^][Tf_2_N^−^] (400 K, 1 bar) in inner and interfacial zones.

	Cation	Anion
[C_4_mim^+^][Tf_2_N^−^] (300 K)		
inner zone		
Confined(CNT(20,20))	28.5_9_	28.3_9_
Confined(CNT(40,40))	4.7_2_	4.5_2_
Confined(SiOH)	0.7_01_	0.5_01_
Confined(Graph.)	0.2_01_	0.2_01_
interfacial zone		
Confined(CNT(20,20))	28.5_9_	28.4_9_
Confined(CNT(40,40))	3.1_3_	3.0_3_
Confined(SiOH)	0.4_01_	0.2_01_
Confined(Graph.)	0.0007	0.0006
[C_8_isoq^+^][Tf_2_N^−^] (400 K)		
inner zone		
Confined(CNT(20,20))	152.4_42_	152.6_44_
interfacial zone		
Confined(CNT(20,20))	152.4_43_	152.6_41_
