# Supplementary material for: Ultrafast diffusion of Ionic Liquids Confined in Carbon Nanotubes
Source: Sci Rep. 2016 Jun 23;6:28518. doi: 10.1038/srep28518 (PMC4917821; doi:10.1038/srep28518)
Supplement: Supplementary Information [file srep28518-s2.pdf]

Ultrafast diffusion of Ionic Liquids Confined in Carbon Nanotubes

Aziz Ghoufi[1,*], Anthony Szymczyk[2], and Patrice Malfreyt[3]

[1]Institut de Physique de Rennes, IPR, CNRS-Université de Rennes 1, UMR CNRS 6251, 35042 Rennes, France.
[2]Institut des Sciences Chimiques de Rennes, UMR 6226 CNRS, Université de Rennes 1, 263 Avenue du Général Leclerc, 35042 Rennes, France
[3]Institut de Chimie de Clermont-Ferrand, ICCF, UMR CNRS 6296, Université Clermont Auvergne, Université Blaise Pascal, BP 10448, 63000 Clermont-Ferrand, France.
[*]aziz.ghoufi@univ-rennes1.fr

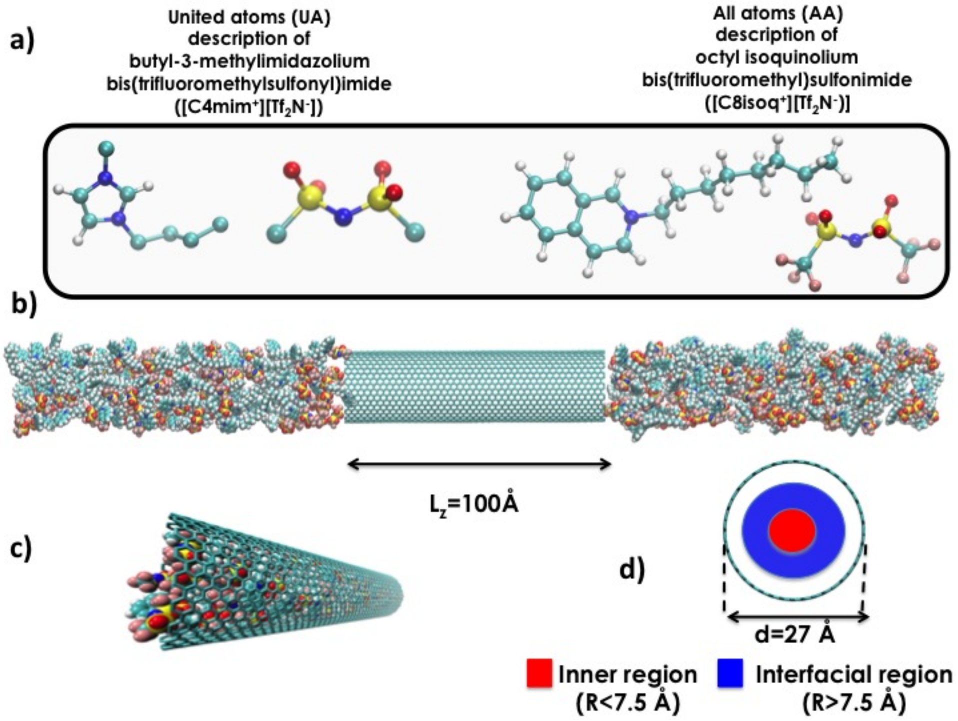

FIG. S1: a)All-atom and united-atom descriptions of [C_4mim$^+$][Tf_2N^-] and [C_8isoq$^+$][Tf_2N^-]). Carbon, oxygen, nitrogen,hydrogen, sulphur and fluorine atoms are in cyan, red, blue, white yellow and pink colors, respectively. b) Illustration of the initial configuration for nanopore filling (with two RTIL reservoirs) c) Snapshot of the production phase (without RTIL reservoirs) d) Definition of the inner and interfacial zones inside a nanopore.

Mean Square Displacement

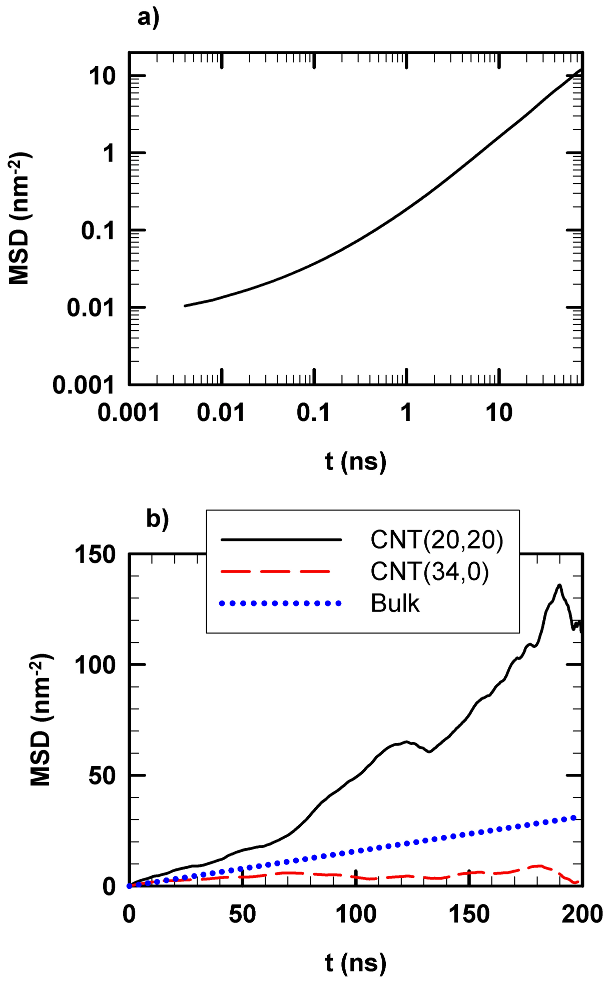

FIG. S2: Mean Square Displacement of [C_4mim^+] center-of-mass at 300 K and 1 bar in a) the bulk phase and b) inside CNT(20,20) and CNT(34,0).

Radial Density

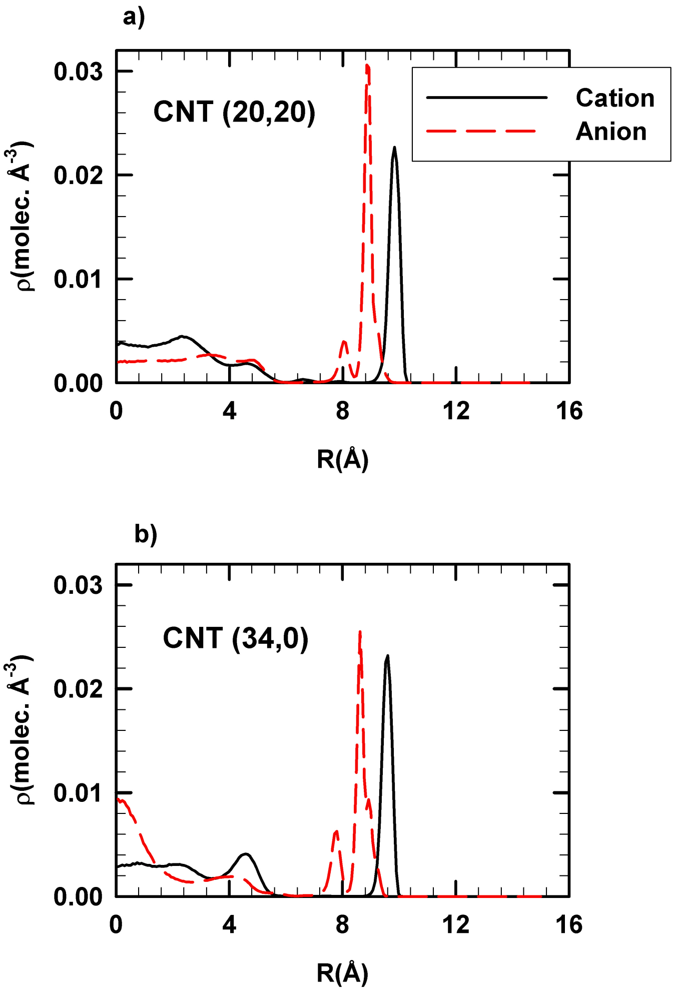

FIG. S3: Radial density of confined [C_4mim^+] and [Tf_2N^-] in a) CNT(20,20) and b) CNT(34,0) at 300 K and 1 bar.

Configuration of [Me_3BuN^+][Tf_2N^-] inside the CNT(20,20)

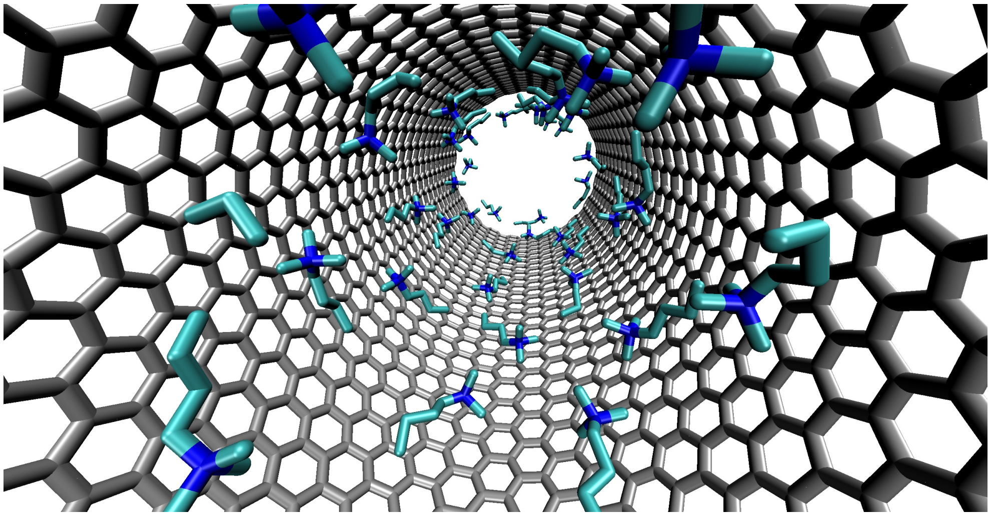

FIG. S4: Snapshot of [Me_3BuN^+] confined inside the CNT(20,20).

Helicoidal Motion

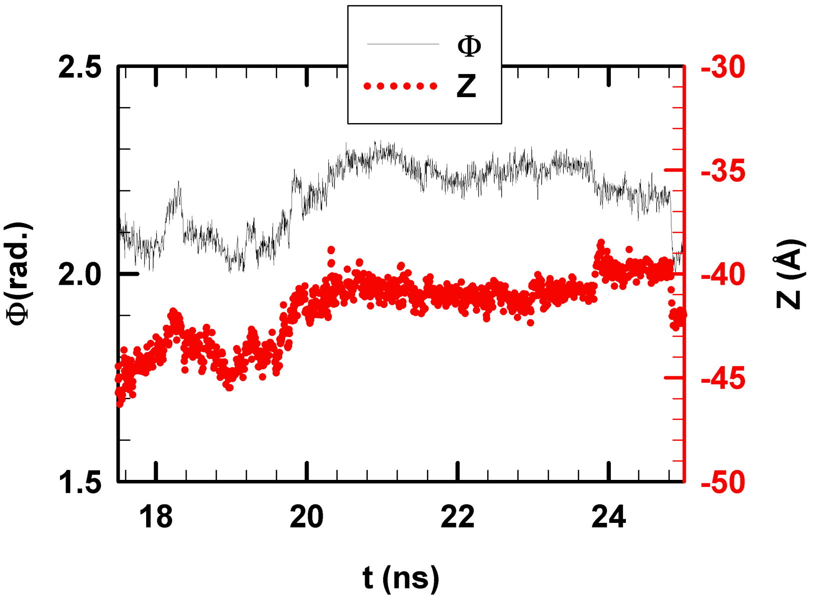

FIG. S5: Time evolution of the azimuthal (black and left axis) and axial (red and right axis) coordinates of a single [C_4mim^+] cation confined in the CNT(40,40).

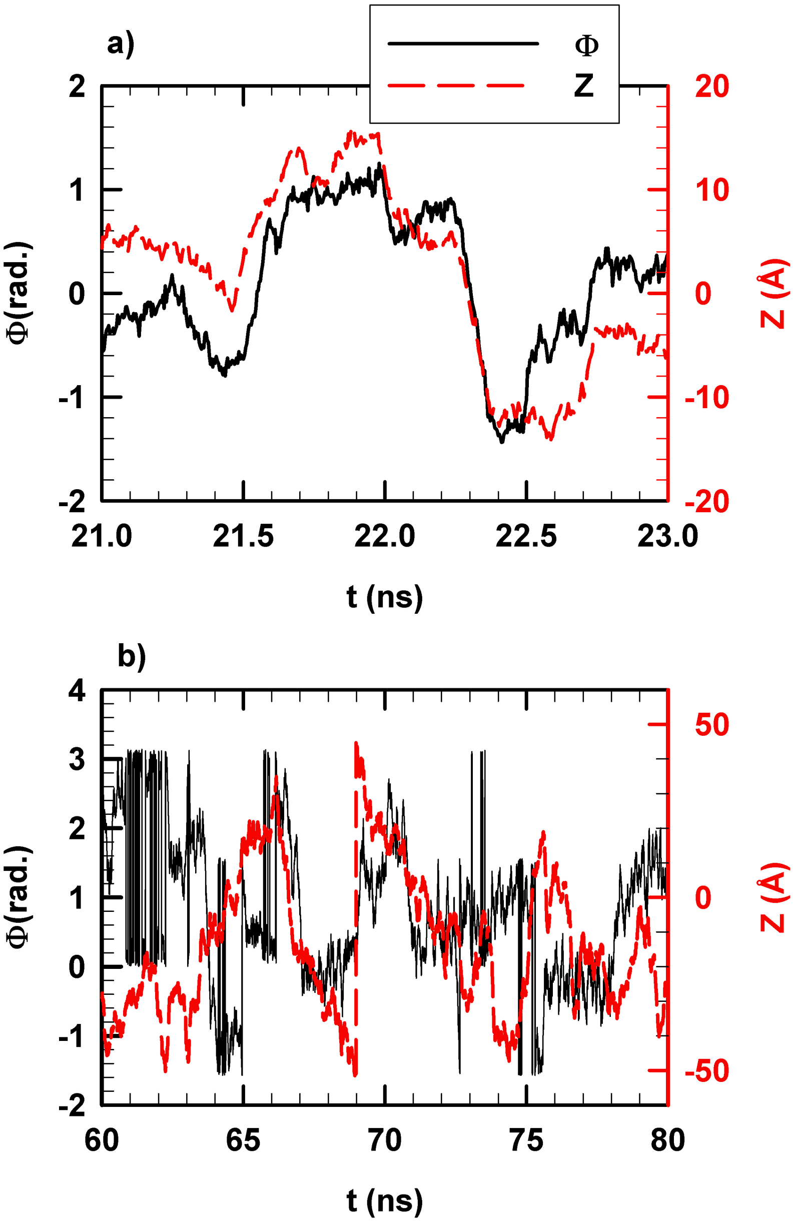

FIG. S6: Time evolution of the azimuthal (black and left axis) and axial (red and right axis) coordinates of a single a) [Me_3BuN^+] cation and b) [C_8isoq^+] confined in the CNT(20,20).

Friction Calculation

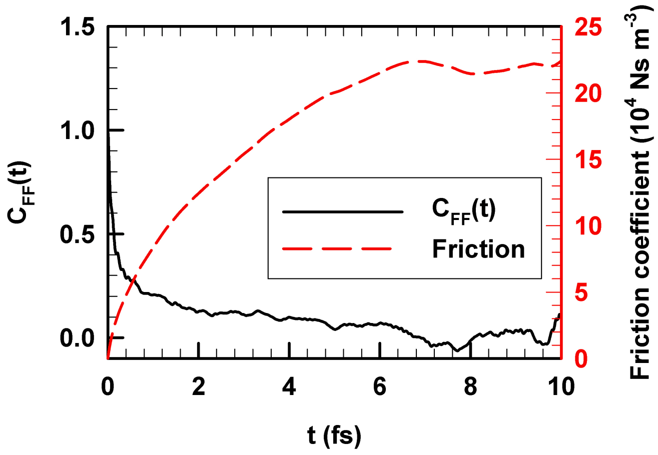

FIG. S7: Correlation function of forces (C_{tt}) and friction coefficient as a function of time (RTIL: [C_4mim^+] [Tf_2N^-]). $C_{tt} = \sum_i^N F_{iz}(t)F_{iz}/F_{iz}(0)^2$ where F_{iz} is defined in the Method Section.

Solvation of ionic liquids

The solvation number was calculated by counting the number of anions within a sphere of radius 8.5 Å from the center of a cation. The distance 8.5 Å was considered since it corresponds to the first valley of the radial distribution function (RDF) between cations and anions. Similar values were found for the various CNTs as well as for the graphene pore.

TAB. S1: Solvation number (N_s^{+-}) of [C_4mim^+] and [Tf_2N^-] confined inside various nanopores at 300 K and 1 bar.

	N_s^{+-}
Bulk(sim.)	5.3
Confined(CNT(20,20))	5.4
Confined(CNT(40,40))	5.4
Confined(CNT(34,0))	5.2
Confined(Graph.)	5.3

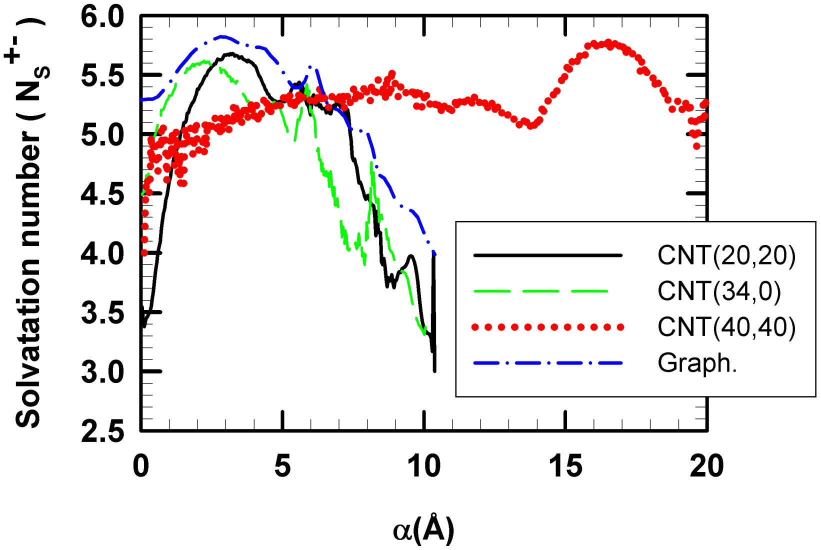

FIG. S7: Solvation number profile(N_s^{+-}) along $\alpha = R$ and $\alpha = Z$ positions inside CNTs and graphene frameworks.

Supplementary Movie

A Movie is provided to highlight the helicoidal motion of [C_4mim^+][Tf_2N^-] within the CNT(20,20).
